# Measurement of finger joint angle using stretchable carbon nanotube strain sensor

**DOI:** 10.1371/journal.pone.0225164

**Published:** 2019-11-14

**Authors:** Jin Woo Park, Taehoon Kim, Daesik Kim, Yongtaek Hong, Hyun Sik Gong

**Affiliations:** 1 Department of Orthopaedic Surgery, Kangwon National University College of Medicine, Chuncheon, Korea; 2 Department of Electrical and Computer Engineering, Inter-University Semiconductor Research Center (ISRC), Seoul National University, Seoul, Korea; 3 Department of Orthopaedic Surgery, Seoul National University College of Medicine, Seoul, Korea; Qatar University, QATAR

## Abstract

Strain sensors capable of monitoring complex human motions are highly desirable for the development of wearable electronic devices and healthcare monitoring systems. Excellent sensitivity and a wide working range of the sensor material are important requirements for distinguishing dynamic human motion. In this study, a highly stretchable strain sensor was fabricated via inkjet printing of single-walled carbon nanotube (SWCNT) thin films on a stretchable polydimethylsiloxane substrate. The sensor was attached to the metacarpophalangeal (MCP) joint of the hand in 12 healthy male subjects. The subjects placed their hands next to a conventional goniometer and flexed the MCP joint to predetermined angles. A linear relationship was found between the change in the length of the strain sensor and the intended angle of the MCP joint. The fabricated thin films showed high durability during repeated cycling (1,000 cycles) and good sensitivity with a gauge factor of 2.75. This study demonstrates that the newly developed stretchable CNT strain sensor can be used for effectively measuring MCP joint angles. This sensor may also be useful for the analysis of complex and dynamic hand motions that are difficult to measure using a conventional goniometer.

## Introduction

Hand therapists record any changes prior to and following treatment by measuring joint angles. Proper measurement of joint movement in daily physical activities over long periods of time is therefore a topic of interest. Three-dimensional (3D) motion analysis systems [1,2] and instrumented gloves have been used for dynamic recording of finger bending angles during the performance of daily activities [3]. Such 3D motion systems can capture complex movements accurately but are bulky and expensive and have large space requirements; they are therefore less suitable for routine clinical use. Instrumented gloves that utilize resistive bend sensors [4] have been developed for application in fields such as computer gaming, virtual reality, rehabilitation, and robotics. Although these gloves are cheaper and easier to set up, they can be cumbersome and require a tedious calibration process; furthermore, the cloth support has been reported to adversely affect measurement performance [5].

Recently, stretchable electronic devices have attracted attention in interesting research areas such as artificial skin, human-interactive devices, and soft robotics [6–8]. Among such devices, a stretchable strain sensor can be considered the most useful owing to its applicability in various fields such as rehabilitation, personal health monitoring, and human motion capture. Flexible and stretchable sensors can be easily embedded into clothing or even attached directly onto human skin. Any applied strain leads to microstructural deformation of the sensor, which, in turn, causes a change in its electrical resistance. The sensor detects resistance changes, and it can thus measure acceleration, pressure, tension, and strain.

Stretching and contraction movements of human joints generate strains as high as 55%, and strain sensors that are capable of detection of such high strains are in high demand [7]. Commercially available strain sensors based on metal foils and semiconductors have extremely poor stretchability (≤5%) and consequently low conformity to biological tissues. Fabrication of stretchable strain sensors with high sensitivity, high durability, fast response, and excellent stability is necessary for the evaluation of human joint motion. For the realization of novel strain sensors that can overcome these issues and meet these requirements, nanomaterials such as carbon nanotubes (CNTs), graphite, and nanowires have been studied. Among such sensors, those based on CNTs have been reported to exhibit excellent electrical and mechanical properties because of the unique molecular structure of CNTs, which makes them suitable for the fabrication of stretchable electronic devices.

Various fabrication methods such as stacking [8], dry-spinning [9], transfer [10], and polymerization of nanocomposites [11–13] have been studied to take advantage of the unique properties of CNTs. For example, Yamada et al. [8] assembled CNT sensors on stockings, bandages, and gloves and demonstrated the ability of the resultant fabricated devices to detect different types of human motion, including movement, typing, breathing, and speech; they therefore concluded that these sensors are promising for use in health care and rehabilitation. However, improvement of the strain sensitivity of CNT-based strain sensors remains a challenge. Stretchable strain sensors composed of CNT films exhibit a piezoresistive response because of the occurrence of a cracking phenomenon under applied strain. Microcracks are formed in a CNT film under stretching, whose density increases with the applied strain and recovers to the initial value after release. The unique structure of CNTs makes CNT films more durable and less sensitive by suppressing crack propagation. Effective and controlled crack generation is necessary for the realization of a highly stretchable and ultra-sensitive strain sensor.

The aim of this study was to fabricate an inkjet-printed CNT strain sensor and evaluate its feasibility for the detection of finger joint motion. The inkjet-printing method provides ease of crack control as well as technical advantages such as precision, ease of patterning, low cost, and large-area scalability. The properties of the fabricated strain sensor were quantified via stretching and cycling strain tests. The excellent properties of the strain sensor enabled detection of finger motion. Because the human hand has multiple measurable joints, we believed that application of the strain sensor to finger joints would be appealing.

## Materials and methods

### Fabrication of strain sensor

**Fig 1:**
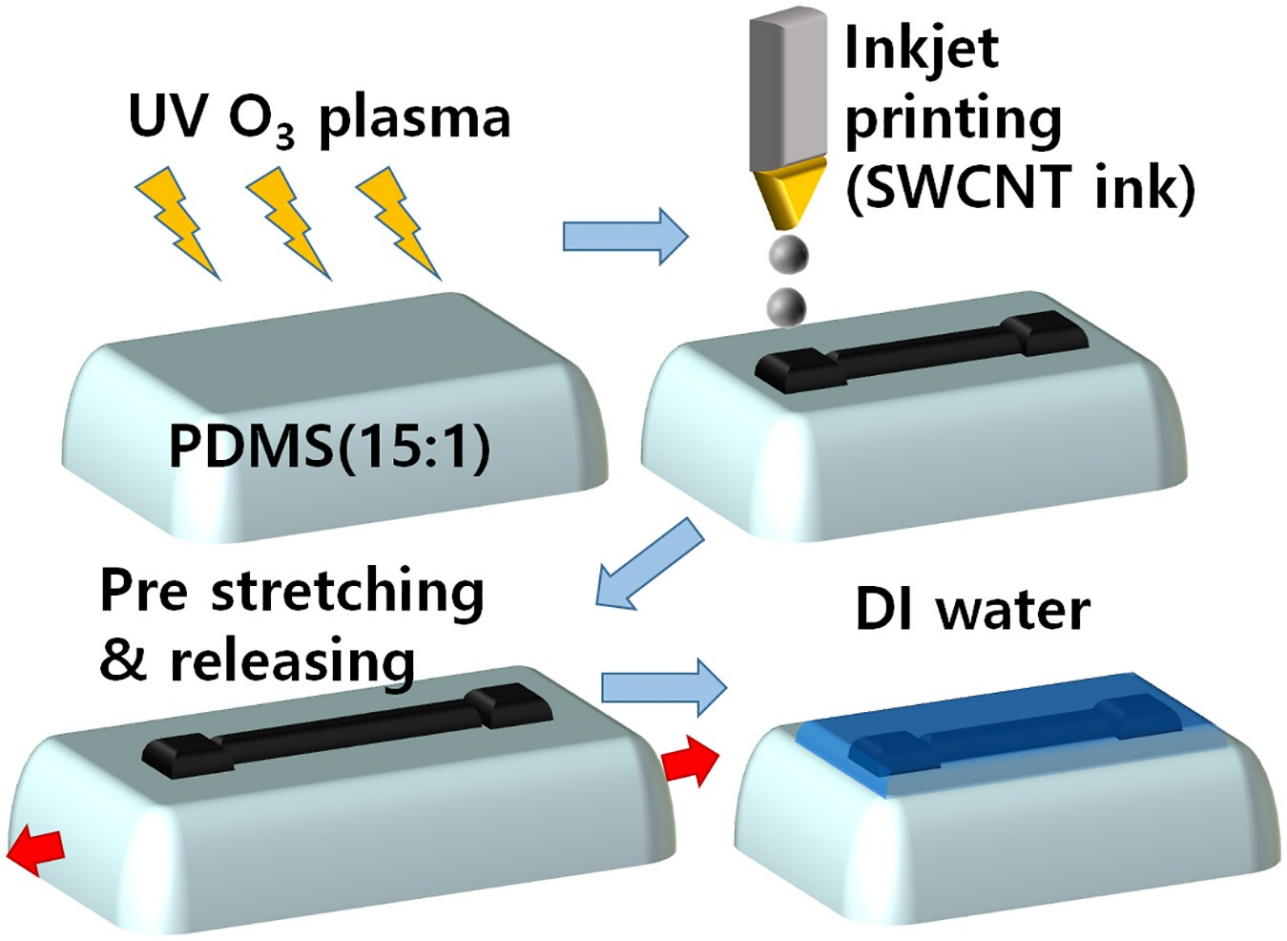
Schematic representation of process used to fabricate inkjet-printed SWCNT thin film on PDMS substrate using aqueous SWCNT ink.

The general process of fabrication of an inkjet-printed CNT thin film has been reported previously [14]. Briefly, aqueous CNT ink was synthesized using a mixture of single-walled CNT (SWCNT)-COOH powder (Fine Chemical Industry, Seoul, South Korea), sodium dodecylbenzenesulfonate (SDBS, Sigma Aldrich, St. Louis, MO, USA), and deionized (DI) water in a weight ratio of 1:5:500. Tip sonication was performed to improve the dispersion stability of the ink. The ink was passed through filter paper to prevent clogging of the printer nozzle. Separately, a stretchable substrate was fabricated by mixing polydimethylsiloxane (PDMS) (Sylgard 184, Dow Corning Corp., Midland, MI, USA) and its curing agent in a weight ratio of 15:1 and then annealing the resulting mixture at 150°C for 15 min. Ultraviolet–ozone treatment of the PDMS substrate was performed so as to improve the wetting properties of the ink. The ink was printed on the surface-treated PDMS substrate in a dog-bone-shaped pattern with two square pads (0.5 × 0.5 cm2) connected by a narrow line (0.1 × 3.0 cm2). In order to induce the formation of numerous cracks in the film, the sample was prestretched with strains of up to 100%. After the prestretching process, DI water was drop-cast on the sample to reduce the amount of SDBS surfactant and consequently the initial resistance of the strain sensor (Fig 1). The sensitivity of the fabricated strain sensor was pretested using one-dimensional automatic stretching devices in order to calibrate measured elongations.

### Measurement of finger joint motion using strain sensor

We measured the angles of the long finger MCP joint in 12 healthy male subjects. This study was approved by the Institutional Review Board of the Seoul National University Bundang Hospital. Subjects provided written informed consent before participation in accordance with the institutional review board’s requirements. The mean age of the subjects was 30 years (age range: 23–35 years).

**Fig 2:**
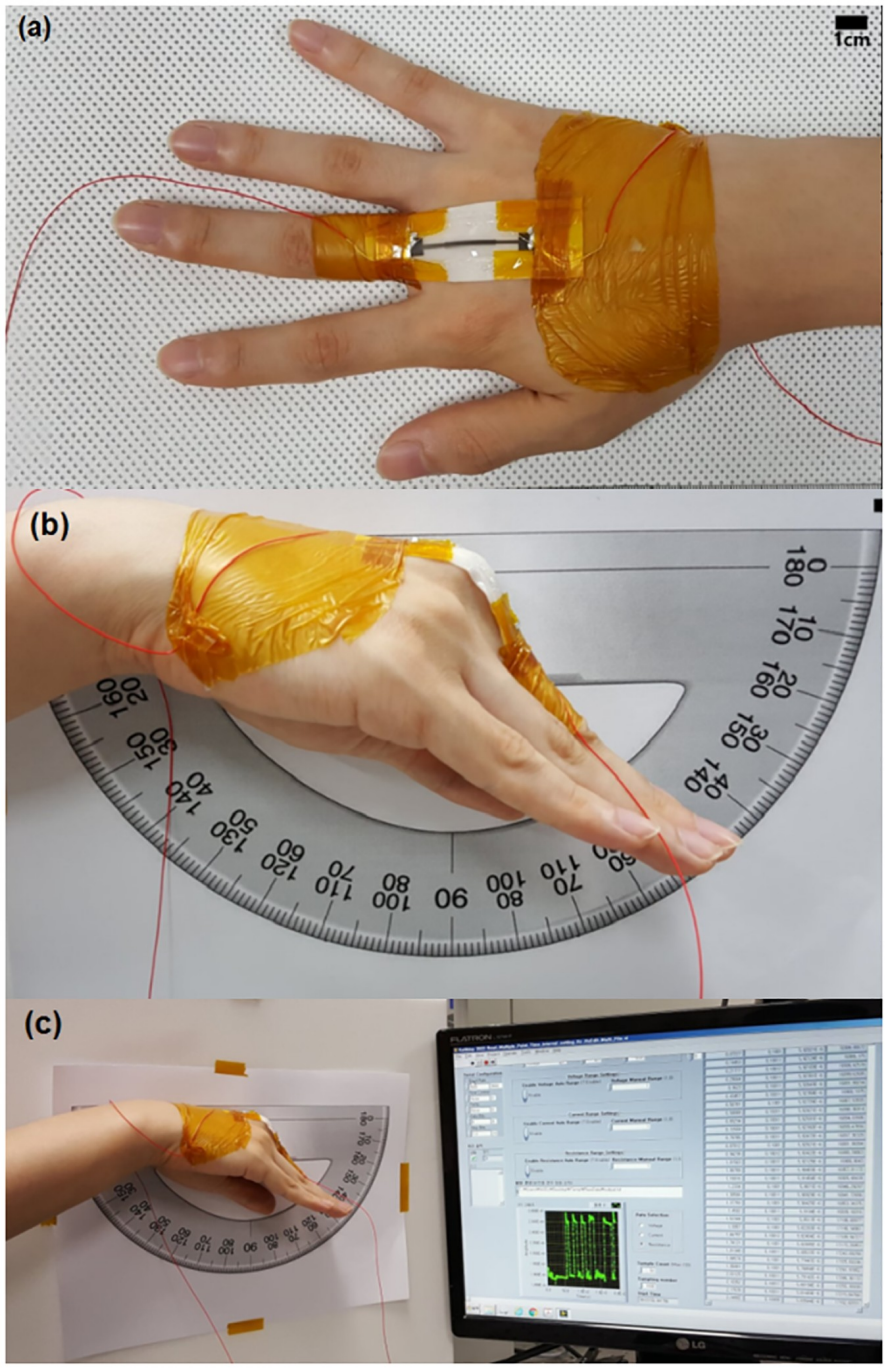
Measurement of finger joint motion using fabricated strain sensor. (a) The 18-mm-long middle segment of the strain sensor was placed directly over the 3rd MCP joint. (b) The hand was placed next to a conventional goniometer (protractor), and the subject was told to flex his fingers at the MCP joint level at predetermined angles of 20°, 45°, 60°, and 90°. (c) A variable resistor was used to measure the change in the resistance between two electrodes during flexion movement of the MCP joint.

Medical adhesive tape was attached to both ends of the strain sensor, which were then fixed over the third MCP joint on the dorsal side (Fig 2a). An 18-mm-long segment at the middle of the strain sensor (hereafter referred to as the initial length) was kept free to measure motion-related strain. Changes in the resistance of the strain sensor were measured using a source meter (Keithley 2400 SourceMeter, Tektronix, Beaverton, Oregon, USA). The MCP joints have two degrees of freedom: one for flexion/extension and the other for abduction/adduction. In order to minimize movement in the coronal plane and thus measure only the flexion/extension angles, a conventional 180° protractor was tacked onto a wall and the subject’s right hand was then positioned such that the radial side of the second finger lay flat on the horizontal axis of the protractor (Fig 2b). The subject was told to flex the MCP joint at predetermined angles of 20°, 45°, 60°, and 90°. The flexion–extension cycle was repeated 20 times for each angle; the subject was told to hold his hand in position for 10 s at the end of each motion (Fig 2c). The subject was then told to repeat the flexion–extension cycle 20 times at random angles. There was a 3-min rest period after each flexion–extension cycle.

The final length of the sensor at maximum flexion for each predetermined angle was calculated using the formula L1 = L0 × (Rb − Ra)/(Ra × gauge factor), where Rb is the resistance at maximum flexion, Ra is the resistance at the initial extended position, L0 is the initial length (18 mm), and the gauge factor represents the sensitivity of the sensor. The gauge factor [(ΔR/R0)/(ε)] of the sensor—where ΔR is the ratio of the resistance change, R0 is the resistance in the initial state, and ε is the applied strain—was calculated beforehand in order to determine the strain sensitivity of the sensor.

## Results and discussion

### Sensor properties under repeated loading–unloading

**Fig 3:**
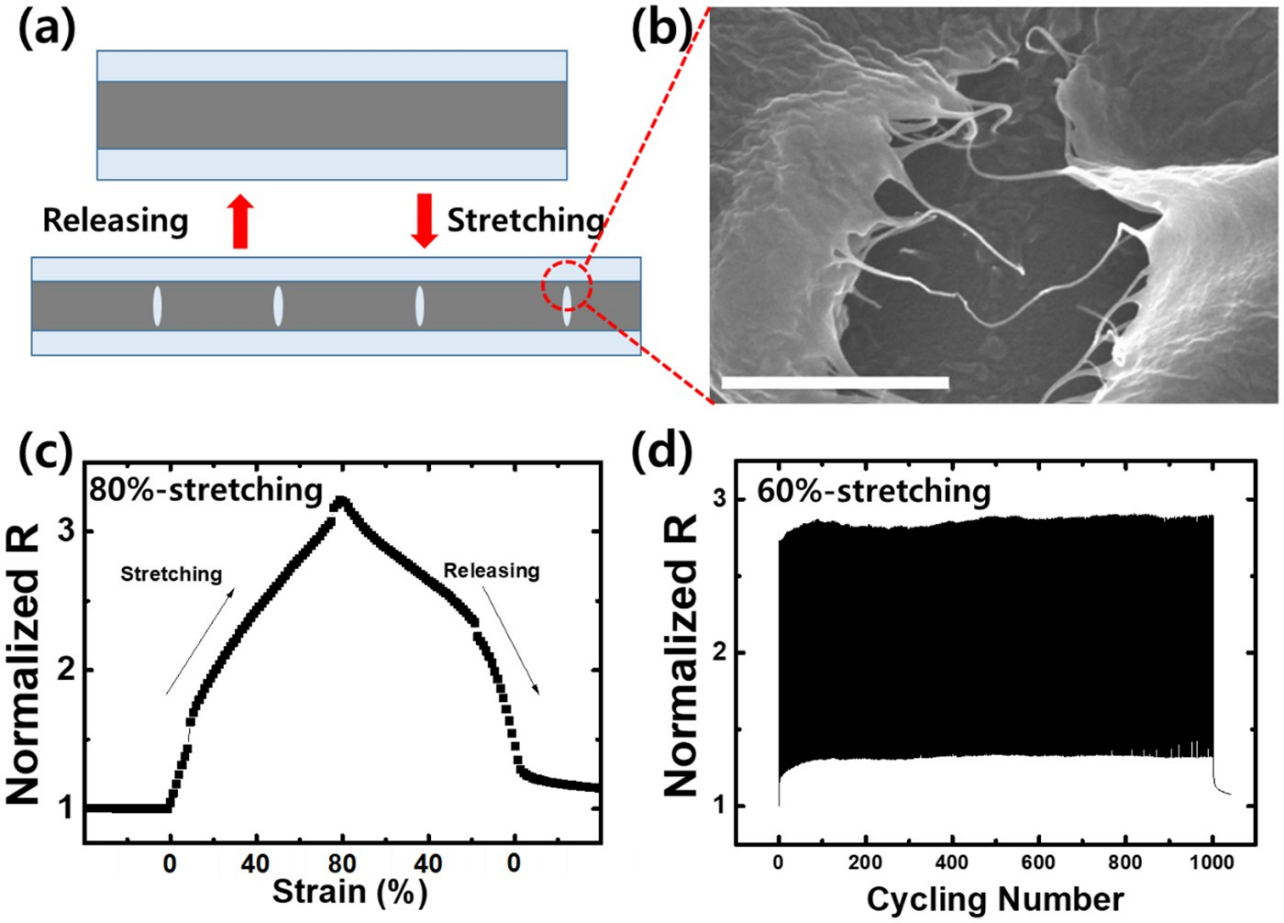
(a) Schematic illustration of SWCNT-PDMS substrate (SWCNT thin film indicated in dark gray), (b) SEM image of SWCNT thin film, (c) relationship between resistance change and time for sensor under 80% strain, and (d) piezoresistive response over 1000 loading–unloading cycles under 60% strain.

**Fig 4:**
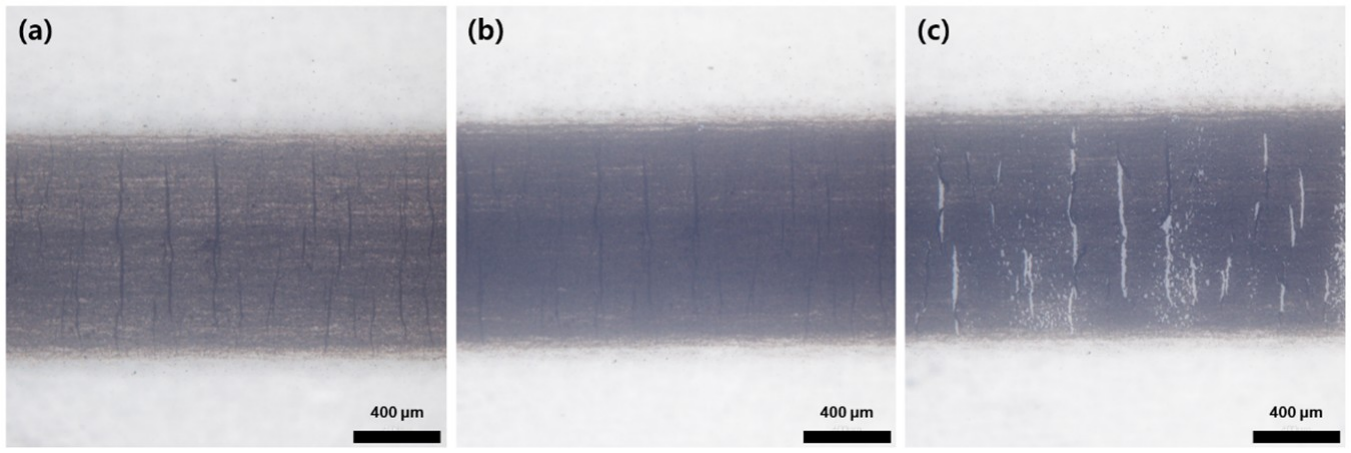
Microstructure of SWCNT strain sensor (a) just after fabrication, (b) after 100 cycles under 80% strain, and (c) after 100 cycles under 120% strain.

As reported in our previous paper [14], the SWCNT film has excellent mechanical properties even under high strains because of a specific phenomenon (crack bridging) that occurs in the film. The printed and subsequently rinsed SWCNT thin film has many cracks that are formed during the fabrication process. These cracks are located mainly in the central part of the film, and they do not propagate to the edge of the film. When an external strain is applied, the cracks widen whereas the conducting path becomes narrow. When the applied strain is removed from the thin film, the conducting path widens, as shown in Fig 3a. During the repeated application of strain, the preformed cracks do not expand easily. As shown in Fig 3b, entanglement is dominant in the SWCNT thin film, which disrupts crack propagation and prevents complete fracture. Because of this intrinsic phenomenon, the SWCNT thin film exhibits good and reliable sensing properties up to 80% strain, as shown in Fig 3c. The gauge factor of the fabricated strain sensor is 2.75. In order to further investigate its durability, strain cycling tests were also performed. Fig 3d reveals a constant variation in the resistance of the sensor. The facile fabrication of the SWCNT thin film together with the occurrence of the above-described intrinsic phenomenon yields a strain sensor that is durable and reliable. We speculate that the high resistances of the SWCNT thin film to microstructural changes prevent its delamination. Therefore, the strain sensor has reliable sensing properties up to 80% strain in an open environment. Under further stretching (>120%), delamination of the SWCNT thin film, which occurs mainly in the preformed cracks, causes deterioration of the reliability of the sensor (Fig 4).

### Environmental effects on sensor performance

**Fig 5:**
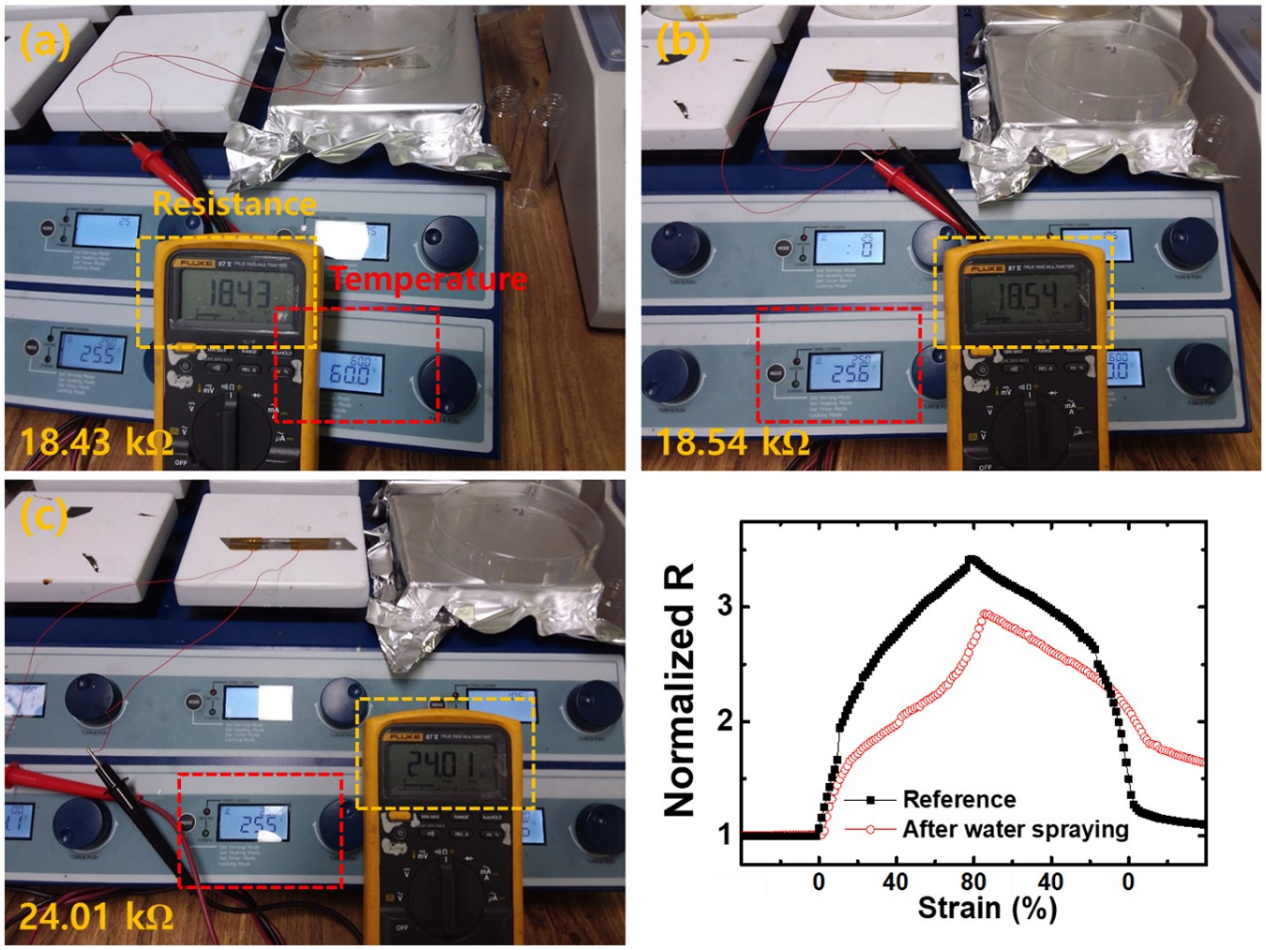
**Initial resistance of the sensor at different states; (a) 60°C, (b) 25°C, (c) 25°C with high humidity**. Resistance values are marked in yellow; temperature values are marked in red. (d) **Resistance variations of SWCNT-based strain sensor under 80% strain (black circles: before water spraying, red circles: after water spraying)**.

In order to investigate environmental effects on the performance of the fabricated strain sensor in a simple manner, its initial resistance in three different states, i.e., reference state (25°C), high-temperature state (60°C), and high-humidity state, was first evaluated. Temperature was controlled using a hot-plate oven and humidity was induced by means of a conventional water spray. As shown in Fig 5a–5c, the temperature effect on the initial resistance was relatively low while the sensor was significantly affected by humidity. The wet sample showed lower and irreversible changes in resistance as shown in Fig 5d. High humidity promoted the delamination of the SWCNT thin film from the substrate, thereby causing an irreversible variation in the resistance. Application of an additional encapsulation layer could be an effective approach to improve the durability of the sensor and to prevent water permeation. However, in our experience, deposition of an additional coating layer onto a crack-based strain sensor significantly lowers its gauge factor. Therefore, we are taking next steps such as developing a patterned substrate to minimize the effects of humidity on the sensor without any degradation in its sensitivity.

### Measurement of finger joint motion using strain sensor

**Fig 6:**
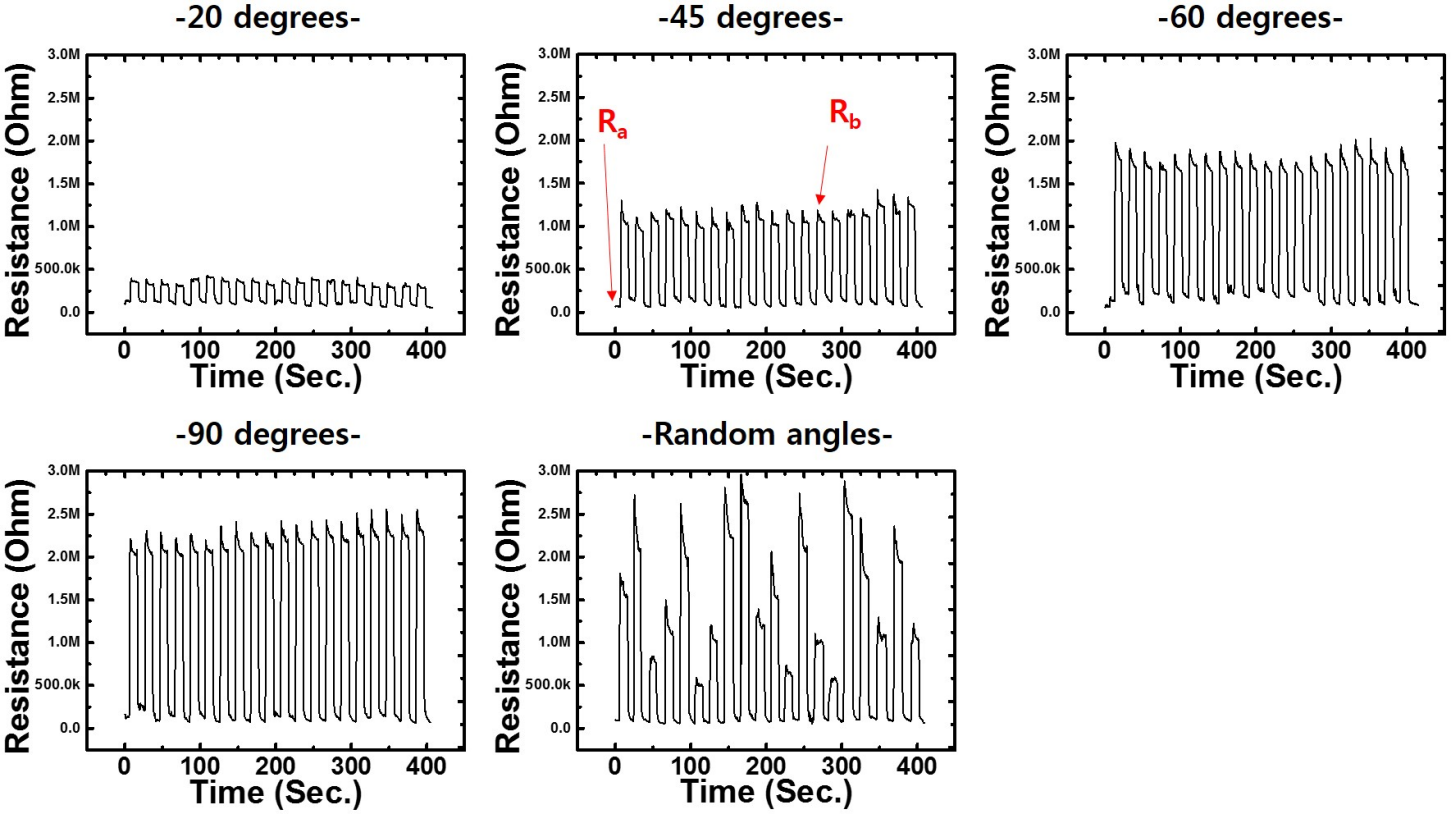
Changes in resistance in various ranges of joint movements. Relative changes in resistance are plotted versus time for the flexion and extension movements of the MCP joint. Here, Ra: initial value of resistance in resting state (neutral position of MCP joint) and Rb: resistance value at predetermined flexion angle.

As shown in Fig 6, the strain sensor responded to the flexion–extension movements rapidly and repeatedly. The high stretchability of the sensor permitted stable elongation (greater than 16 mm in extreme flexion) throughout the flexion–extension cycles. When the subject bent his finger at various angles, the sensor generated resistance signals with different intensities because larger bending angles resulted in greater elongations of the sensor and an increase in its resistance. The resistance of the sensor remained constant when the finger was held at a certain angle for 10 s, which demonstrates the excellent stability of the sensor. The resistance returned to the original value when the finger was restored to the neutral position.

### Linearity of strain-sensor-measured angles

**Fig 7:**
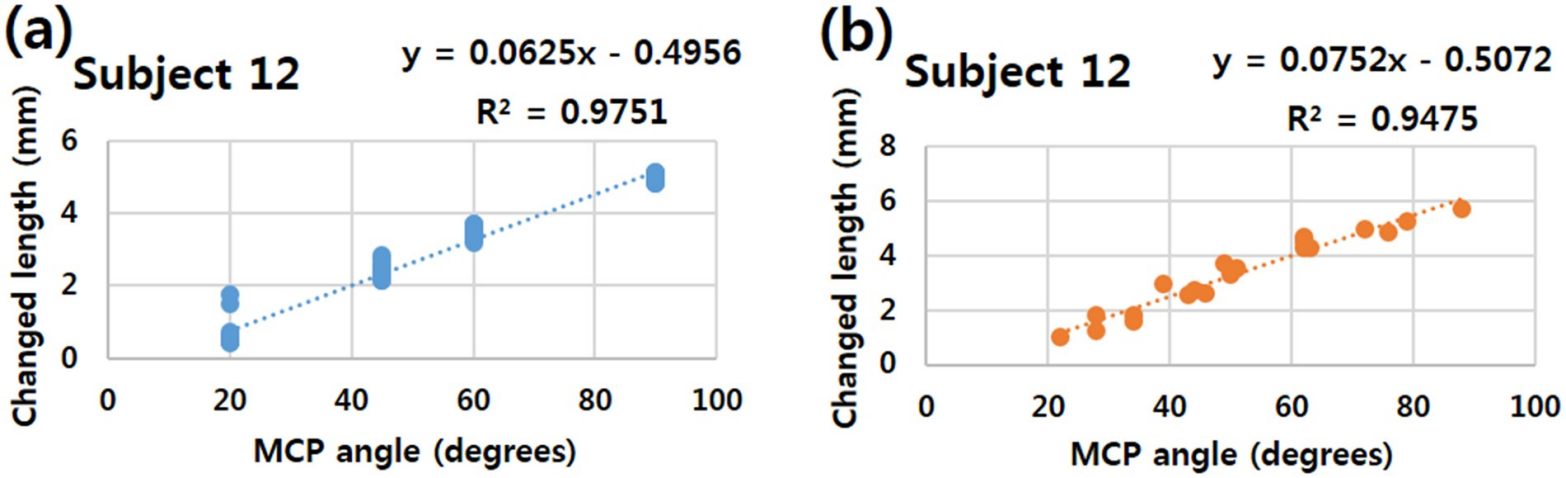
Relationship between change in sensor length and angle of MCP joint for subject no. 12 shown as an example. (a) High linearity of the correlation (R2 = 0.9751, p = 0.000) between the MCP joint flexion angle (x-axis) and the change in the length of the strain sensor (y-axis) is observed when the finger is flexed 20 times each at 20°, 45°, 60°, and 90°. (b) High linearity of the correlation (R2 = 0.9475, p = 0.000) between the MCP joint flexion angle and the change in the length of the strain sensor is also observed when the 3rd MCP joint is flexed 20 times at random angles between 0° and 90°.

The linearity of the relationship between the change in the length of the sensor and the angle of the MCP joint for each subject was analyzed by simple regression analysis. The MCP joint angle was the independent variable and the change in the length of the strain sensor was the dependent variable. A highly linear relationship was found between the change in the length of the strain sensor and the intended angle of the MCP joint (Fig 7). The R2 values of each subject ranged from 0.713 to 0.975, indicating a high degree of linearity. The linear increase in the sensor length with increasing bending angle indicates the ability of the sensor to detect and quantify the applied strain.

Measurement of joint motion is crucial for diagnosing musculoskeletal and neuromuscular disorders, as well as for monitoring disease progression or assessing treatment efficacy. The universal goniometer is commonly used in evaluations of major joints; however, there are doubts about its inter-tester reliability and any measurement performed using a goniometer has a potential maximum error of 10° [15]. Evaluation of the motion of hand joints is especially important since maintaining motion is essential for precise tasks, and it is associated with patient satisfaction after orthopedic interventions [16]. However, conventional goniometric measurement of finger joints can be affected by factors such as tissue edema, goniometer arm length, and adjacency of fingers, and it generally requires more precise handling than that of larger joints [17]. Conventional static measurements are often not reflective of actual functional capacity, and they need to be further complemented by dynamic evaluation [4].

Strain sensors with high stretchability and high sensitivity are desirable for full-range detection of human motions. However, conventional metal or semiconductor sensors usually have low sensitivity or narrow sensing ranges. Nanomaterials such as CNTs, graphene, and metallic nanowires have been used to address these issues. CNTs, in particular, have been widely applied as stretchable electrodes in various fields because of their excellent mechanical properties. The present study shows that a CNT-based strain sensor with high stretchability and high sensitivity is able to monitor small changes in length at the finger joint level and that there is good agreement between goniometric measurements and the actual elongation of the strain sensor.

Wearable strain sensors should have high performance in terms of their stretchability, sensitivity, linearity, and durability. In this study, we selected CNT films because they show highly homogenous microcrack propagation and lateral interconnections; furthermore, cracks formed upon stretching maintain their connections even after release [18]. The high stretchability of CNTs is attributed to the reversible opening and closing of gaps. The maximum strain generated by human joint movement (as high as 55%) is typically much higher than the detection limit of conventional strain gauges [19]. Inkjet-printed SWCNT thin films have previously been demonstrated to maintain excellent conductive properties under 100% tensile strain. Only very small increases in resistance have been reported during high strain cycling tests [14]. PDMS elastomer is widely used in microfabrication because it is capable of forming any structure and is biocompatible; additionally, it can maintain excellent contact with human skin during natural movements [20]. Because of the flexibility and stretchability of our fabricated sensor, it can be easily mounted onto human skin with the aid of medical adhesive tape.

Inkjet printing was adopted in this study because it offers unique advantages over other printing methods. For example, it facilitates rapid printing at low cost and enables precise control of the amount of deposited material. Furthermore, multiple layers can be easily deposited on top of each other, which aids realization of highly conductive films of CNTs [21].

There are some limitations to this study. The study sample size was small, and the study was limited to one joint of the hand. Furthermore, only flexion and extension movements were studied. However, despite these limitations, our obtained findings for healthy hands are expected to be useful in the development of wearable sensors for the diagnosis and rehabilitation of joint problems in orthopedic patients. Such sensors could be useful for pediatric patients with congenital deformities of the extremities, because their joints are small and it is difficult for children to follow instructions during measurement of the active range of motion. Our highly sensitive strain sensor is also expected to be useful in measurements aimed at analysis of continuous dynamic motion for patients with neuromuscular disorders such as cerebral palsy.

## Conclusions

In conclusion, a SWCNT/PDMS strain sensor was fabricated by inkjet printing, which is a cost-effective, additive method. The sensor was mounted onto human skin without discomfort. The fabricated sensor showed high stretchability as well as a highly linear relationship between the intended flexion angle and the change in length. This sensor, if refined further and put into practice, may be useful for analyzing complex and dynamic hand motions that are difficult to measure using a conventional goniometer.
